# Nonlinear Structured Illumination Using a Fluorescent Protein Activating at the Readout Wavelength

**DOI:** 10.1371/journal.pone.0165148

**Published:** 2016-10-26

**Authors:** Hui-Wen Lu-Walther, Wenya Hou, Martin Kielhorn, Yoshiyuki Arai, Takeharu Nagai, Michael M. Kessels, Britta Qualmann, Rainer Heintzmann

**Affiliations:** 1 Leibniz Institute of Photonic Technology, Jena, Germany; 2 Institute of Biochemistry I, Jena University Hospital/Friedrich Schiller University Jena, Jena, Germany; 3 The Institute of Scientific and Industrial Research, Osaka University, Osaka, Japan; 4 Institute of Physical Chemistry, Abbe Center of Photonics, Friedrich-Schiller-University Jena, Jena, Germany; University of California Berkeley, UNITED STATES

## Abstract

Structured illumination microscopy (SIM) is a wide-field technique in fluorescence microscopy that provides fast data acquisition and two-fold resolution improvement beyond the Abbe limit. We observed a further resolution improvement using the nonlinear emission response of a fluorescent protein. We demonstrated a two-beam nonlinear structured illumination microscope by introducing only a minor change into the system used for linear SIM (LSIM). To achieve the required nonlinear dependence in nonlinear SIM (NL-SIM) we exploited the photoswitching of the recently introduced fluorophore Kohinoor. It is particularly suitable due to its positive contrast photoswitching characteristics. Contrary to other reversibly photoswitchable fluorescent proteins which only have high photostability in living cells, Kohinoor additionally showed little degradation in fixed cells over many switching cycles.

## Introduction

In the past few years, the ongoing development of super-resolution imaging techniques has spurred cell biological research world-wide, as previously unresolvable details of cellular structures became visible. Among these methods, structured illumination microscopy (SIM) draws attention to fast super-resolution imaging, as this method does not only provide a two-fold resolution improvement with respect to the Abbe limit, but also stands out by its potential for fast acquisition [1–4]. Thus SIM potentially allows exploring even fast dynamics and functions in living cells and tissues. Therefore, SIM has been successfully used in various biological studies based on imaging. By using a programmable spatial light modulator (SLM) rapid control of the structured illumination pattern is achieved [3–7].

Other than improving the acquisition rates of SIM and working in thick specimens [2, 5,8], another goal is to push the resolution of SIM beyond the factor of two (compared to the Abbe limit) as present in linear SIM. This can be achieved by introducing a nonlinear photo-response of the fluorescence emission to the intensity of the excitation light. Saturating the excited state of fluorophores is a possibility to create the desired nonlinearity [9–12]. However, driving the excited state into saturation requires very high illumination intensity, which usually leads to unacceptably high photo-bleaching and photo-toxicity. Such high intensities can be avoided if longer-lived states are used. A particularly attractive variant is the use of switching between chemical states [13] that can be driven into saturation. Reversibly photoswitchable fluorescent proteins (RSFPs) are thus suitable for super-resolution at low light intensities achieving a resolution of 50–100 nm. This approach has been termed RESOLFT (reversible saturable optical fluorescence transitions) [14]. Depending on the detailed characteristics of the photoswitching, there are various ways to achieve the desired nonlinear response. Table 1 presents the characteristics of Kohinoor and other RSFPs. Typical RSFPs, such as Dronpa, rsEGFP, Skylan-S, Skylan-NS, have negative photoswitchability, meaning exciting at the readout wavelength causes deactivation. Kohinoor and Padron are recently developed RSFPs with positive photoswitchability, meaning excitation at the readout wavelength causes activation whereas a separate wavelength is used for deactivation.

**Table 1:** Characteristics of Kohinoor, Padron, Dronpa, rsEGFP, Skylan-S and Skylan-NS. The values were taken from literature, ref. [15–18]. Brightness is represented as a percentage with respect to that of Dronpa. N/A: no data available. *: Brightness of Skylan-S and Skylan-NS is a relative value to Dronpa calculated from values given in ref. [15] and [18], respectively.

	Positive RSFPs		Negative RSFPs			
	Kohinoor	Padron	Dronpa	rsEGFP	Skylan-S	Skylan-NS
Maximum absorption (nm)	495/386	503/396	503/392	493	499	499/386
Maximum emission (nm)	514	522	522	510	511	511
Quantum yield	0.71	0.64	0.68	0.36	0.64	0.59
Brightness	67	41	100	25	115*	92.8*

## Acquisition Method in NL-SIM

In the past years, Dronpa used to be a favorite candidate of the RSFPs in NL-SIM experiments [11, 12]. Although it offers high quantum yield, it requires special embedding media for fixed samples, its relaxation time is long and its photostability is low, leading to bleaching after only a few switching cycles. More recently, rsEGFP attracted a lot of interest due to its high photostability allowing for >1,200 switching cycles [19]. However, its quantum yield as well as the number of photons yield per cycle is low causing low signal-to-noise ratios (SNRs). Low SNRs are a major problem for NL-SIM data reconstruction.

**Fig 1:**
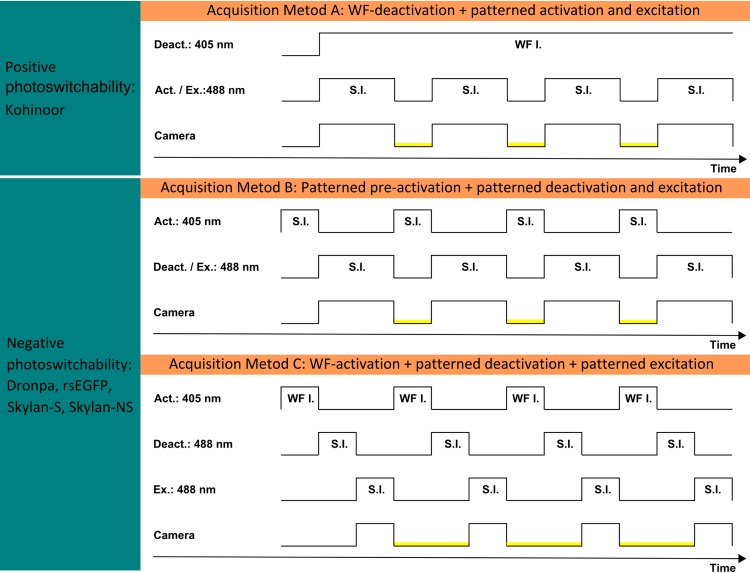
NL-SIM acquisition methods comparison between fluorophores with positive contrast photoswitching characterics and fluorophores with negative contrast photoswitching characterics. Three different trigger timings of synchronization for NL-SIM experiments are present. Note that all triggers are on the low-to-high edge. The region marked in yellow indicates the timing of the camera readout. S.I.: structured illumination. WF I.: wide-field illumination. A) NL-SIM acquisition with Kohinoor. Kohinoor is deactivated with wide-field illumination at 405 nm and is activated and excited with structured illumination at 488 nm. B) NL-SIM acquisition with negatively switching RSFPs. A pre-activation with structured illumination at 405 nm is required. Then a structured illumination at 488 nm with a phase shift of π is used to acquire NL-SIM raw data. This method requires very precisely adjusted patterns switching between 405 nm and 488 nm illuminations over a large field of view. C) Further scheme for negative switching RSFPs. Pre-activation with uniform illumination at 405 nm followed by structured illumination at 488 nm to deactivate most of the fluorophores. A π phase shifted structured illumination at 488 nm with π is used to read out the signal from the remaining fluorophores. This scheme avoids precise achromatic matching between two patterns.

Recently, Li *et al*. [3] presented a major break-through by conducting NL-SIM imaging with newly developed RSFP Skylan-NS and achieved a resolution of ~45 nm. However, the use of Skylan-NS, which suffers from the same negative photoswitchability as the other RSFPs, is currently either limited by the need of using violet light for patterned activation (see Fig 1 Acquisition Method B) or requires a more sophisticated acquisition method (see Fig 1 Acquisition Method C). A violet laser at 405 nm wavelength was used for patterned activation followed by a patterned readout of the RSFP Skylan-NS in [3]. However, producing a perfect illumination pattern that is precisely aligned to the illumination pattern of the 488 nm laser at every position is challenging. Moreover, the 405 nm laser is not permitted for operation of the SLM by its manufacturer and extended use may damage the device. The other alternative of doing NL-SIM with Skylan-NS is to employ a sophisticated acquisition method: Wide-field activation at 405 nm laser followed by a patterned depletion at 488 nm and successive patterned readout phase shifted by π to the depletion step. Although alignment issues can thereby be avoided, the trade-off is a complicated and longer acquisition (see Fig 1 Acquisition Method C) and loss of signal.

**Fig 2:**
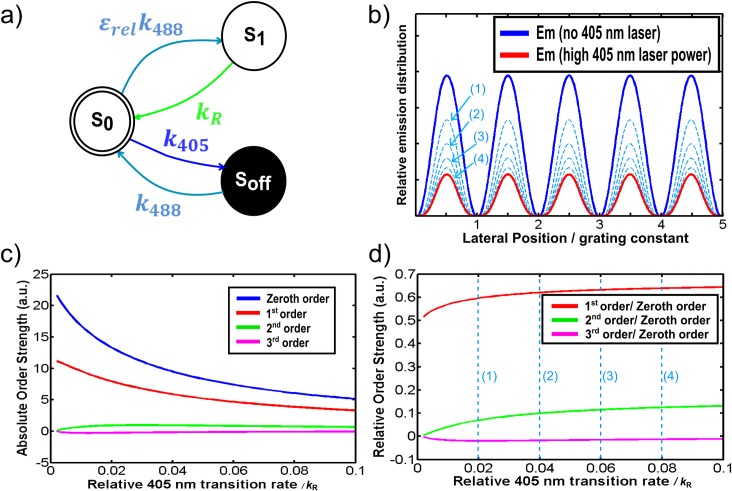
Illustration of switching behavior of Kohinoor and simulation of fluorescent emission distribution in a two-beam NL-SIM scheme. a) Our state diagram of Kohinoor used in the simulations. Excitation and photo-activation at 488 nm, deactivation at 405 nm. S_off_, S_0_, S_1_ and *k*_R_ denote the non-fluorescent dark state, the fluorescent ground state, the fluorescent excited state and the fluorescence emission rate, respectively. b) Simulated dependence of the relative lateral fluorescent emission in a steady state situation without photo-deactivation (blue solid line) and with deactivation at under strong 405 nm illumination (red solid line) at a relative transition rate of 0.1*K*_R_ in the maximum. The blue dashed lines of (1) to (4) correspond to relative deactivation rates of 0.02*k*_R_, 0.04*k*_R_, 0.06*k*_R_ and 0.08*k*_R_ respectively. c) Absolute component strength in dependence of relative deactivation rate. d) Component strength normalized to the total intensity. The blue dashed lines (1) to (4) correspond to the dashed lines in panel b.

Here we demonstrate that some of these limitations can be overcome by a NL-SIM setup using the newly developed fast-switching fluorescent protein Kohinoor [16]. The excitation maximum of Kohinoor is at 495 nm and its emission maximum is at 514 nm. The most useful characteristic of Kohinoor is its positive photoswitchability, meaning it can be activated and read out at 488nm whereas it is deactivated at 405nm (Fig 2A) [16]. To avoid the complication of achromatic alignment we chose to demonstrate its applicability to NL-SIM imaging using a steady state approach with a patterned activation and readout competing with a uniform depletion at 405nm. This means that both light sources, 405 nm and 488 nm, were illuminating the sample simultaneously during camera exposure. During the camera readout, the 488 nm laser was off and the 405 nm laser remained on to “clear” the scene by switching remaining fluorophores to the dark state (see Fig 1 Acquisition Method A). Since in contrast to the use of Skylan-NS the 405 nm laser was not applied in a spatially patterned way to generate the nonlinear fluorescent emission response, it was not necessary to send the 405 nm laser light to the SLM. Our NL-SIM design therefore protected the SLM from the potentially destructive 405nm. A benefit is that our data acquisition method is exactly as in linear SIM (LSIM) configurations [6]. Although Padron also offers positive photoswitchability, a disadvantage of Padron is that violet light also excites fluorescence emission which is problematic in our acquisition scheme [17, 20].

## Principle of NL-SIM

**Fig 3:**
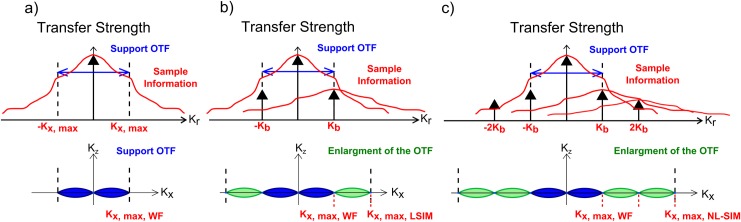
LSIM and NL-SIM Principle. a) Fourier transform of sample emission generated under uniform illumination. Sample information in Fourier space lying inside the region of support of the OTF, defining a cut-off frequency of K_x,max_, is detectable. b) Fourier transform of linear sample emission under sinusoidal illumination (LSIM). In addition to the zero frequency as in a, two ±1^st^ object components at ±K_b_ are generated. Hence high-frequency sample information is downshifted into the support region of the detection OTF. Accordingly, the effective OTF (lower part) is enlarged. c) Fourier transform of sample emission under nonlinear structured illumination conditions. The nonlinear sample response introduces higher illumination harmonics in Fourier space to all of which the sample information is attached.

In an optical system, the optical transfer function (OTF) defines the maximum transferable spatial frequency. High frequency information outside the passband of the OTF is lost and thus fine specimen details cannot be seen. In 2-beam LSIM, fluorophores are illuminated with a sinusoidal illumination pattern shifted to the positive range, referred to as the illumination grating. In Fourier space this corresponds to three delta peaks in the illumination causes a local emission proportional to the illumination intensity and the local fluorophore density attaching the object to each illumination order in Fourier space and summing them up. The detected image in the image plane corresponds to its convolution with the point spread function of the image system, i.e. to a multiplication with the optical transfer function in Fourier space. By unmixing these three components and shifting each of the three object component (Fig 3B) back in Fourier space such that the zero object frequency position coincides with the zero frequency position of Fourier space, the effective OTF is expanded. High-frequency object information outside the passband of the original OTF is now within the support region of the detection OTF and a superresolved image with two-fold resolution improvement can be reconstructed.

In NL-SIM the sample emission is assumed to remain linear with respect to the sample fluorophore concentration but nonlinear with respect to the illumination intensity. This allows the nonlinearity to be seen as an “effective excitation” pattern (also called “emittability”). A sinusoidal illumination thus causes a non-sinusoidal effective excitation in NL-SIM. This can be achieved by saturating transition states of the fluorophores. The non-sinusoidal effective excitation pattern contains higher-order harmonics that all carry the same object information in Fourier space. Consequently, separating object information from higher-order harmonics and rejoining them at their correct object position frequency space yields, in principle, infinite resolution. Practically, however, the resolution remains affected by the saturation level and only the frequency orders above the noise level are distinguishable from the noise. These two aspects, nonlinearity and signal to noise ratio, are key limiting parameters for the final NL-SIM resolution (Fig 3C).

In this work, we demonstrate an experimental design that achieves NL-SIM imaging using RSFP Kohinoor. We activated and excited Kohinoor with an illumination grating at 488 nm and deactivated Kohinoor under uniform illumination at 405 nm. In the steady state approximation, the populations of three transition states of the fluorophores, that is dark state <*S*_*off*_>, fluorescent ground state <*S*_0_> and excitation state <*S*_1_>, are constant. The time derivative of the population of each state is zero.

$$0=\frac{\partial <S_{off}>}{\partial t}=k_{405}<S_{0}>-k_{488}<S_{off}>$$ (1)

$$0=\frac{\partial <S_{0}>}{\partial t}=k_{488}<S_{off}>-k_{405}<S_{0}>-\varepsilon_{rel}k_{488}<S_{0}>+k_{R}<S_{1}>$$ (2)

$$0=\frac{\partial <S_{1}>}{\partial t}=\varepsilon_{rel}k_{488}<S_{0}>-k_{R}<S_{1}>$$ (3)

$$1=<S_{0}>+<S_{off}>+<S_{1}>$$ (4)

with *k*_R_ representing the rate constant of fluorescence. *k*_405_ and *k*_488_ are the transition rates of the fluorophores affected by the 405 nm and the 488 nm laser, respectively. Both *k*_405_ and *k*_488_ are proportional to the laser intensity and are functions of the lateral position in the specimens and the grating phase. As the 488 nm laser activates and excites fluorophores simultaneously, we introduced *ε*_*rel*_ as the relative activation efficiency. By using Eq (1) and Eq (2), Eq (4) can be rewritten as

$$1=\frac{k_{R}}{\varepsilon_{rel}k_{488}}<S_{1}>+\frac{k_{405}k_{R}}{\varepsilon_{rel}k_{488}^{2}}<S_{1}>+<S_{1}>$$ (5)

The fluorescent emission distribution can then be expressed as

$$k_{R}<S_{1}>=\frac{k_{R}\varepsilon_{rel}k_{488}}{k_{R}(1+\frac{k_{405}}{k_{488}})+\varepsilon_{rel}k_{488}}$$ (6)

At low excitation levels far below singlet state saturation, the dominant term in the denominator is the constant *k*_*R*_. This means, we can simplify the system by assuming *ε*_*rel*_ to be unity as it contributes predominantly an overall sample brightness effect, i.e. no change in the general behavior is expected at illumination intensity far from singlet excited state saturation. In the steady state, the emission pattern is no longer sinusoidal and higher-order harmonics are created (Fig 2B). The order strength of the zeroth, the 1^st^, the 2^nd^ and the 3^rd^ order was calculated from the Fourier transform of each effective excitation distribution assuming steady state conditions for a range of relative 405-induced transition rates (Fig 2C). The relative component strengths of the 1^st^, the 2^nd^ and the 3^rd^ order were plotted normalized to the order strength of the zeroth order, i.e. the overall brightness (Fig 2D). In our steady state NL-SIM, the sample is illuminated at both 405 nm and 488 nm, to simultaneously cause activation, excitation and deactivation. Since 405 nm illumination also reduces the overall emission (see the zeroth order in Fig 2C) there will be a trade-off in terms of the useful range of 405nm illumination (see also Fig 2B). Only the first higher-order harmonics (±2^nd^ component orders) appears sufficiently strong to be detectable above the noise level (Fig 2D). Besides, the noise limits the possible resolution-enhancement and thus the effective OTF cannot be expanded arbitrarily. Only signal that can be discriminated from noise contributes to the high-resolution object information in a reconstructed image. Noise, especially in low-SNR photo-switching situations, is known to severely affect the SIM reconstruction [3]. Although there certainly is room for improvement by using a better switching scheme, the acquisition method is yet interesting as it is as simple as in LSIM and we utilized the camera readout time of 2.494 ms for fluorophore deactivation under uniform wide-field illumination at 405 nm. Note that steady state conditions as modeled in our work are not a requirement and may even be a disadvantage, but they ease the modelling and allow for an optimization of the experimental parameters such as the integration time vanishes from the equations.

**Fig 4:**
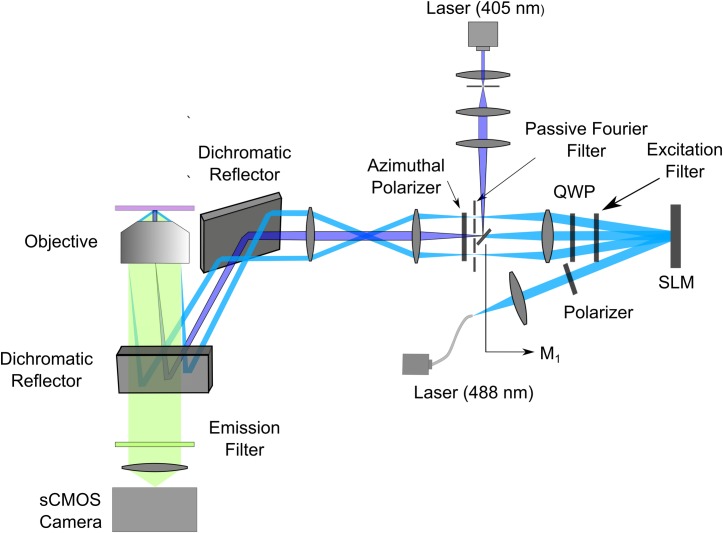
Fast nonlinear structured illumination microscope setup. A 488 nm laser was coupled into a single-mode polarization-maintaining fiber for activation and excitation of the fluorophores. A polarizer ensured linear polarization of the light before the SLM. The fast ferroelectric liquid crystal SLM was limited to displaying binary patterns. An excitation filter was used for cleaning up fluorescence generated by the SLM. A quarter wave plate (QWP) together with the azimuthal polarizer ensures azimuthal polarization of the light for all grating orientations to guarantee high contrast of the grating pattern in the sample. The zero diffraction order of the 488 nm laser was blocked by the backside of a very small mirror (M_1_) used to reflect the 405 nm laser into the system near the Fourier plane of the SLM for uniform illumination. The ±1^st^ diffraction orders of the 488 nm laser passed through a passive filter at the Fourier plane of the SLM. Both 405 nm and 488 nm lasers were sent to the sample plane through a 63× oil immersion microscope objective (NA 1.46, alpha Plan-Apochromat, Korr TIRF, Zeiss, Germany). Fluorescence was imaged and collected through an emission filter by the sCMOS camera (Orca Flash 4, Hamamatsu, Japan).

Our NL-SIM system was adopted from the two-beam fastSIM setup, described in [6] as depicted in Fig 4. A small mirror (see Fig 4, M_1_) was used to integrate the 405 nm laser into the system in the Fourier plane of the SLM. The 405 nm laser can be focused to one of the triangular linear polarizers of an azimuthally patterned polarizer resulting in a uniform illumination of the specimen. The grating patterns, which were generated with the grating search algorithm presented in ref. [6], were displayed on the SLM (SXGA-3DM, 1280 × 1024 pixels, Forth Dimension Displays, UK) and projected into the specimens. The average grating constant was ~181.3 nm. The illumination grating was rotated to six orientations, separated by ~30° to achieve near isotropic resolution improvement. In order to derive high-resolution object information from the ±1^st^ and ±2^nd^ component orders, for each grating orientation, five images at phases 0, 2π/5, 4π/5, 6π/5 and 8π/5 were first recorded and then the grating was rotated to the next orientation. In total, 30 raw images were used for the reconstruction of one NL-SIM image.

## Sample Preparation and Cell Line Information

**pcDNA3-Kohinoor-Actin** [16] was amplified using the NucleoBond® Xtra Midi/Maxi kit (MACHERY-NAGEL GmbH & Co. KG).

**HeLa cells** were maintained in DMEM medium (Gibco® Life technologies) supplied with 10% fetal bovine serum, 0.1 mM gentamicin and 1× penicillin (100 units/ml)-streptomycin (100 μg/ml) and cultured at 37°C under 5% CO_2_.

**For transfection and fixation**, almost confluent HeLa cells were seeded into a 6 well plate and transfected 24 h later following the user manual of the TurboFect transfection reagent (Thermo Scientific, R0531). After 48 h, transfected HeLa cells were seeded (1:3 dilution) onto high precision microscope cover glasses (18 × 18 mm) and cultured for another 24 h. Afterwards, cells were fixed with 1 ml pre-warmed 4% (w/v) PFA for 15 min at 37°C. After washing with PBS, the coverslips were mounted upside down onto slides using Mowiol^®^ 4–88 (Calbiochem), dried overnight at room temperature and stored at 4°C.

## Results and Discussion

### Photoswitching Test on Kohinoor

**Fig 5:**
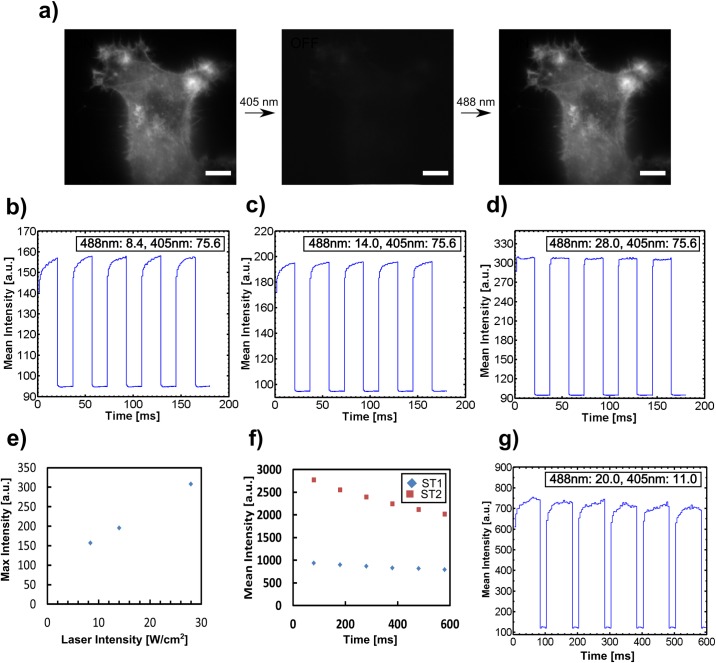
Repeated photoswitching test on Kohinoor-actin expressed in a fixed HeLa cell. a) A fixed HeLa cell expressing Kohinoor-actin was switched on and excited by a 488 nm laser. A 405 nm laser was used to switch the fluorophores off. Scale bar: 5 μm. Photoswitching of Kohinoor over time with the light intensity of 8.4 W/cm^2^ (488 nm) + 75.6 W/cm^2^ (405 nm), b) 14.0 W/cm^2^ (488 nm) + 75.6 W/cm^2^ (405 nm), c) 28.0 W/cm^2^ (488 nm) + 75.6 W/cm^2^ (405 nm), d) respectively. e) The maximum fluorescence intensity of the 20^th^ raw image in b), c) and d). f) The mean fluorescence intensity in the last raw image of each excitation section with 488 nm irradiation over six switching cycles. ST1: 14 W/cm^2^ (488 nm) + 27 W/cm^2^ (405 nm) and ST2: 20 W/cm^2^ (488 nm) + 32 W/cm^2^ (405 nm), respectively. g) Photoswitching of Kohinoor at 20.0 W/cm^2^ (488 nm) + 11.0 W/cm^2^ (405 nm). The mean intensity denotes fluorescence emission intensity. The units are consistent in all plots.

We first tested the photo-switching of Kohinoor on fixed HeLa cells transfected with Kohinoor-actin using alternating uniform irradiation at 405 nm and 488 nm (Fig 5). In the first part of the photo-switching experiment, we acquired 20 images, each with effectively 1 ms of 488 nm laser illumination followed by deactivation with the 405 nm laser for ~16 ms in each switching cycle. Three intensity combinations of 488 nm and 405 nm at the sample were tested, 8.4 W/cm^2^ (488 nm) + 75.6 W/cm^2^ (405 nm) (Fig 5B), 14 W/cm^2^ (488 nm) + 75.6 W/cm^2^ (405 nm) (Fig 5C) and 28 W/cm^2^ (488 nm) + 75.6 W/cm^2^ (405 nm) (Fig 5D), respectively. The results show that higher intensities of the 488 nm laser saturated the on-state faster also inducing more fluorescence. Fig 5D shows that on-state saturation happened immediately when the high intensity of 488 nm was present. Specifically the relationship between 488 nm excitation intensity and maximum fluorescence emission intensity appears to be linear, see Fig 5E.

**Fig 6:**
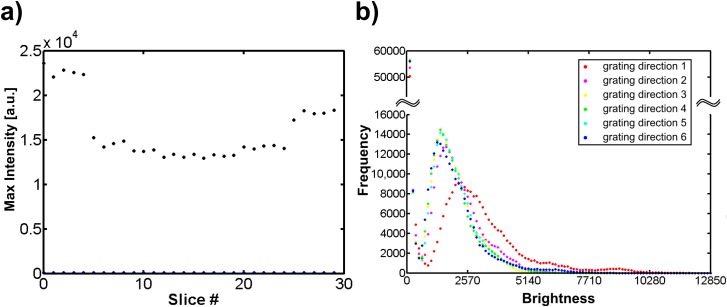
Brightness of Kohinoor. a) The value of the brightest pixel of each image in all 30 acquired raw images was calculated. 30 dark images were acquired for calculating the background value. b) Brightness histogram of six raw images in six grating directions.

In the second part of the photo-switching experiment, we acquired 20 images, each with effectively 4 ms of 488 nm laser illumination followed by deactivation at 405 nm for ~20 ms in each switching cycle. Two light intensity combinations for 488 nm and 405 nm at the sample were tested, ST1: 14 W/cm^2^ (488 nm) + 27 W/cm^2^ (405 nm) and ST2: 20 W/cm^2^ (488 nm) + 32 W/cm^2^ (405 nm), respectively. Fig 5F shows the fluorescence intensity over time. In Fig 5F, we selected a longer excitation exposure time compared to Fig 5B–5D and the higher excitation intensity induce more photo-bleaching as is seen by comparing the ST1 and ST2 curves. However, photo-bleaching turned out to be minor for our NL-SIM acquisition as the fluorescence intensity was still sufficient for image reconstruction after a total acquisition time of ~0.52 second (see Fig 6).

In the third part of the photo-switching experiments (Fig 5G), we reduced the 405 nm deactivation intensity to 11 W/cm^2^ and acquired 20 images, each with effectively 4 ms of 488 nm laser illumination (20 W/cm^2^) followed by deactivation at 405 nm for ~20 ms in each switching cycle. We observed that at a low 405 nm intensity, fluorophores deactivated effectively as the fluorescence intensity curve in each switching cycle shows a clear increasing tendency.

In the following NL-SIM experiment, laser intensities of 56 W/cm^2^ + 11 W/cm^2^ at the sample for the 488 nm and the 405 nm lasers, respectively were finally selected for carrying out fast saturation and high nonlinearity of the emission pattern.

We imaged the actin filaments in 2D with our two-beam NL-SIM microscope with the SIM pattern under nearly total internal reflection conditions. The exposure time of structured illumination at 488 nm for fluorophore activation and excitation in each raw image was 12 ms. The 405 nm laser was switched on 0.22 seconds before the acquisition started and was kept on until the end of acquisition. While the camera read out each raw image, the 405 nm illumination depleted residual on-state fluorophores for ~2.494 ms (see Fig 1 Acquisition Method A). All 30 raw images were acquired in ~0.52 seconds as needed for one final 2D NL-SIM image reconstruction. The pixel size in the reconstructed NL-SIM image corresponds to 21.5 nm.

The averaged value of the brightest pixel in all 30 acquired raw images is 19231.5. The averaged background value calculated from 30 dark images is 94.3. Fig 6A shows the value of the brightest pixel in each acquired raw images (black dots) and the background value in each dark images (blue dots). Fig 6B presents the histogram of brightness of the first raw image in each grating direction. The results show that the fluorescence intensity remains high and sufficient for NL-SIM image reconstruction after a total acquisition time of ~0.52 second without being affected much by the photobleaching.

**Fig 7:**
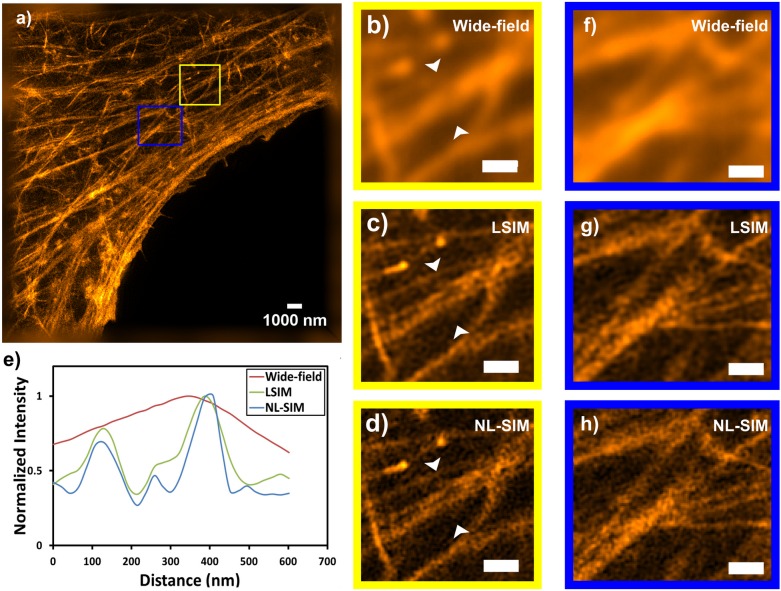
NL-SIM imaging of a fixed HeLa cell expressing Kohinoor-actin. a) NL-SIM image in a large field of view. Magnified view of the yellow-boxed region corresponds to a conventional wide-field microscopy image b), LSIM image c), and NL-SIM image with one extra component d). e) Normalized intensity profiles between the white triangles in b)-d). Magnified view of the blue-boxed region represent conventional wide-field microscopy image f), LSIM image g), and NL-SIM image h). Scale bar in magnified images is 500 nm.

In the wide-field image obtained by summing all raw images, the fine F-actin network cannot be resolved (Fig 7B and 7F), linear SIM already yielded high resolution images (Fig 7C and 7G). Reconstructing images from NL-SIM with one extra harmonic increased the resolution marginally (Fig 7D and 7H). The actin filaments became less blurry and more discrete. Fig 7E shows the normalized intensity profiles between the white triangles which is analyzed by ImageJ, using bilinear interpolation to retrieve intensity values along oblique line selections. (Rasband, W.S., ImageJ, U. S. National Institutes of Health, Bethesda, Maryland, USA, http://imagej.nih.gov/ij/, 1997–2016)

In LSIM and NL-SIM, the underlying assumption is that the sample always behaves predictably. However, in the case of our steady state approach, the light used for activation, deactivation and excitation of Kohinoor are simultaneously present. In stark contrast to localization-based methods such as dSTORM, the single-molecule switching deviates from the assumption of a continuous sample ignoring molecules and molecular states. If only one or just a few molecules contribute to a pixel and undergo molecular switching, leads to significant artefacts in the SIM reconstruction, even if many photons can be detected from a single fluorophore. The NL-SIM reconstruction model assumes a sample at a certain position to contribute for example 20% of its full brightness during readout when it is switched off with 80% chance beforehand. However, a single molecule will be in either of the two states, “on” or “off” and thus either contribute 100% or 0% brightness during readout. Such effects average out either by many molecules contributing to the pixel or by the molecules undergoing many switching cycles while contributing to the measured results. Nevertheless this super-Poissonian statistics can be expected to contribute to the artefacts in the final image, even if many photons can be detected form a single fluorophore. Since the concentration of fluorophores is not very far from the single-molecule regime, we addressed this problem by opting for several switching cycles contributing to a single detected image by applying both activation and deactivation simultaneously. This greatly simplifies the acquisition configuration and also prevented our image acquisition from drowning in the noise of molecular switching but a disadvantage of this choice is that the achievable level of nonlinearity remained relatively low (see Fig 2D). The potential for a stronger nonlinearity is however limited with this scheme. The resolution we obtained in NL-SIM was improved only marginally beyond the two-fold-beyond-Abbe-limit resolution gain as obtained in LSIM.

## Conclusion

We confirmed the high photostability, photoswitchability and strong fluorescence of Kohinoor in fixed HeLa cells. We used a positively RSFP Kohinoor for NL-SIM imaging based on the steady state approach but the concession is the low nonlinearity and thus a limited resolution improvement. The attainable acquisition speed in fast NL-SIM is well suited to follow fast dynamics in biological samples at enhanced resolution compared to the diffraction limit.

Our NL-SIM approach leaves room for improvement. In order to fully exploit the shown advantages of the NL-SIM set-up with Kohinoor as RSFP for analyses of molecular structures and dynamics in cells, future work will have to focus on computationally accounting for the effect of single-molecule switching. Solving this problem in the future would further increase the resolution improvement achieved beyond standard SIM. A direction of future research to improve the super-resolution method further may be iterative schemes with preparation steps (activation, depletion) interleaved with detection ideally all contributing to the same camera exposure. This may be achievable with very fast mechanical shutters or choppers.
